# Phyto-Assisted Assembly of Metal Nanoparticles in Chitosan Matrix Using *S. argel* Leaf Extract and Its Application for Catalytic Oxidation of Benzyl Alcohol

**DOI:** 10.3390/polym14040766

**Published:** 2022-02-16

**Authors:** Amel Taha, Enshirah Da’na

**Affiliations:** 1 Department of Chemistry, College of Science, King Faisal University, P.O. Box 400, Al-Ahsa 31982, Saudi Arabia; ataha@kfu.edu.sa; 2 Department of Biomedical Engineering, College of Engineering, King Faisal University, P.O. Box 400, Al-Ahsa 31982, Saudi Arabia

**Keywords:** cross-linked chitosan, biosynthesis, gold nanoparticles, *Solenostemma argel*, catalytic oxidation

## Abstract

The design and synthesis of eco-friendly solid-supported metal nanoparticles with remarkable stability and catalytic performance have gained much attention for both industrial and environmental applications. This study provides a novel, low-cost, simple, and eco-friendly approach for decorating cross-linked chitosan with gold nanoparticles (AuNPs), greenly prepared with *Solenostemma argel* (*S. argel*) leaf extract under mild conditions. Glutaraldehyde-modified chitosan beads were used to coordinate with Au(III) ions and act as stabilizing agents, and *S. argel* leaf extract was used as a cost-effective phyto-reducing agent to reduce gold ions to elemental Au nanoparticles. The successful cross-linking of chitosan with glutaraldehyde, the coordination of Au(III) ions into the chitosan matrix, and the phytochemical reduction of Au(III) to Au nanoparticles were investigated via FT-IR spectroscopy. The obtained Au nanoparticles have a uniform spherical shape and size <10 nm, as confirmed by both X-ray diffraction (XRD) (~8.8 nm) and TEM (6.0 ± 3 nm). The uniformity of the AuNPs’ size was confirmed by Scanning Electron Microscopy (SEM) and Transition Electron Microscopy (TEM). The powder X-ray diffraction technique showed crystalline AuNPs with a face-centered cubic structure. The elemental analysis and the Energy Dispersive Spectroscopy (EDS) analysis both confirmed the successful integration of Au nanoparticles with the chitosan network. The catalytic activity of this highly stable nanocomposite was systematically investigated via the selective oxidation of benzyl alcohol to benzaldehyde. Results showed a remarkable conversion (97%) and excellent selectivity (99%) in the formation of benzaldehyde over other side products.

## 1. Introduction

Nowadays, the design and synthesis of inorganic/organic hybrid materials (metal nanoparticles/polymer) have attracted much attention, due to their important applications in biomedical [1,2], sensor [3,4,5], semiconductor [6], catalysis [7,8,9,10], solar cell [11], and many other fields [12]. This wide range of applications is mainly due to the fact that these hybrid nanocomposites have attractive electrical [13], optical [13,14,15], and magnetic [16,17,18] characteristics compared to those of pure polymers or inorganic particles. Many polymers, such as poly (amidoamine) (PAMAM) [19,20,21,22] and PPV [11], have been utilized as stabilizing agents or support for different metal nanoparticles, such as copper [23], gold [24], silver [25], and platinum [26].

One of the most abundant biopolymers is chitosan, which is derived from the distillation of chitin that occurs naturally in the exoskeletons of insects, crustacean shells, and fungi cellular walls [27]. Both chitin and chitosan are biocompatible, biodegradable, and non-toxic, with antimicrobial and hydrating effects [27,28]. However, chitosan is preferred, because of its higher solubility [27]. Chitosan-based hybrid materials are considered effective bio-sorbents, since they are relatively cheap and their surfaces are rich in amino and hydroxyl groups [29,30]. They were reported to significantly capture many pollutants, such as p-nitrophenol [31] and heavy metal ions [27]. Furthermore, they have a high chelating affinity towards metal ions, which is related to the high content of nitrogen (6.89%) [29]. Accordingly, chitosan can function as the ideal support for many different species, such as ions and molecules [28,29,32,33]. However, the main challenge to the application of this biological support is its high solubility in most organic acids and dilute mineral solutions. This drawback can be overcome by chemical or physical modification via cross-linking agents, such as ethylene glycol diglycidyl ether, glutaraldehyde (GLA), epichlorohydrin, genipin, or triphosphate [31,34,35]. Among these agents, GLA is the most attractive, since it does not alter the adsorption capacity of chitosan [31]. In addition to enhancing chemical stability, this cross-linking can improve both the mechanical and thermal stability of the chitosan [34,35].

Many synthesis routes have been reported in the literature for AuNPs supported on chitosan derivatives [36,37,38,39,40]. However, there is still serious concern about these routes, mainly related to harsh synthesis conditions, high cost, complexity, the need for toxic chemicals, and metal leaching problems. Furthermore, these approaches are not attractive for biological applications, since they may be toxic and non-biocompatible. Thus, there is a need to develop eco-friendly and green techniques without the need for any toxic chemicals or harsh conditions. So far, many biological species, such as bacteria, viruses, fungi, and plant extracts, have been utilized for the synthesis of AuNPs [41,42,43]. Among all of these facilitating species, plant extract has many attractive features, such as scalability, economic feasibility, simplicity, availability, and non-toxicity [41,44,45]. Carlo et al. (2012) reported a green synthesis route to combine gold nanoparticles with chitosan network via using different organic acids to control the rate of reduction of AuIII into AuNPs and the morphology of the resulting nanoparticles [30].

*Solenostemma argel* (Apocynaceae) is a North African, naturally grown desert plant that is widely available in Libya, Sudan, Egypt, Algeria, and Saudi Arabia [46]. It is widely used to traditionally treat many diseases [47,48]. The chemical composition of *S. argel* leaves have been reported in the literature [49]. El-Zayat et al. reported the presence of antifungal, antioxidant, and antibacterial components in the leaf extract [48]. Another chromatographic investigation of *S. argel* leaves revealed the presence of many chemical components, such as flavonoids and alkaloids, which are known to have excellent reduction and capping effects [50]. 

Based on this, it is expected that *S. argel* leaf extract can function as a green reducing agent. Thus, combining it with cross-linked chitosan may enhance the phytochemical assembly of metal nanoparticles within the organic matrix. Accordingly, this current work reports a novel green phytochemical route for the bioreduction of Au(III) ions to elemental Au atoms with the aid of *S. argel* leaf extract, instead of a chemical reducing agent, for the synthesis of gold nanoparticles within the chitosan matrix under very mild conditions. To the best of our knowledge, the utilization of cross-linked chitosan and leaf extract in green preparation of metal nanoparticles has never been reported. Furthermore, the structural properties of the resulted hybrid nanocomposite and its catalytic performance towards the degradation of alcohol were investigated. 

## 2. Experimental

### 2.1. Materials

All chemicals and solvents were used as received without further purification. Hydrogen tetrachloroaurate trihydrate (HAuCl_4_.3H_2_O) was obtained from Merck (Kenilworth, NJ, USA); chitosan flakes, glutaraldehyde 50%, benzyl alcohol, and 4-nitrophenol were purchased from Sigma Aldrich (Missouri, MS, USA). All aqueous solutions were made using deionized water. *S. argel* leaves were purchased from the local market of Hofuf City, Eastern Province, Kingdom of Saudi Arabia.

### 2.2. Methods

#### 2.2.1. Preparation of *S. argel* Leaf Extract

To prepare the *S. argel* leaf extract, 20 g of the dried and ground leaf was mixed with 100 mL of deionized water in a 500 mL round flask for one hour. After that, the mixture was boiled for 20 min and filtered under a vacuum, then collected and stored at 4 °C.

#### 2.2.2. Cross-Linked Chitosan Preparation

The chitosan solution was prepared by mixing 2 g of chitosan flakes with 60 mL of acetic acid solution (5% *v*/*v*) and stirring for 18 h. Then, the remaining acid within the chitosan gel was neutralized to coagulate the chitosan gel into uniform spherical beads. This was achieved by dropwise precipitation of the mixture into 250 mL of a 0.5M NaOH bath under magnetic stirring at 100 rpm. After that, the beads were thoroughly rinsed with distilled water until neutral pH was reached, dried under ambient conditions, ground, and sieved through a mesh size <200 µm. A suspension was then prepared by mixing a specific mass of the chitosan beads with a similar mass of 2.5% GLA solution for 24 h at 100 rpm and 20 °C. The chitosan-GLA beads were then separated by filtration and thoroughly rinsed with hot distilled water followed by cold distilled water. Finally, the beads were dried, ground, and sieved as before. During the synthesis process, different colors were noticed, indicating some chemical changes taking place during each step. 

#### 2.2.3. Preparation of Chitosan-GLA/AuNPs Hybrid Nanocomposite

A specific mass of the synthesized chitosan-GLA beads (0.5 g) was mixed with 30 mL of 0.3 mM HAuCl_4_ aqueous solutions and stirred for a few minutes to allow the adsorption of Au(III) ions on the chitosan-GLA beads; then, it was filtered, thoroughly washed with deionized water, and dried. To reduce Au(III) into Au nanoparticles, 30 mL of *S. argel* leaf extract was mixed with 0.5 g of chitosan-GLA beads loaded with Au(III) ions and stirred for 18 h. Finally, the Au-chitosan-GLA beads were filtered, washed with deionized water, dried in a vacuum, and then stored in a dry place.

#### 2.2.4. Characterization Methods

FT-IR spectra of chitosan beads after each step of synthesis were recorded with a Cary 630 FT-IR spectrophotometer within the range of 400 to 4000 cm^−^^1^. UV–vis absorption spectroscopy analysis was performed in a Shimadzu UV-1800 spectrophotometer (Kyoto, Japan) using quartz cuvettes. Spectra were collected within the range of 200–800 nm. The crystalline nature of the Au particles in chitosan matrix was analyzed by X-ray diffraction on an XRD Bruker (Billerica, MA, USA) D8 advanced diffractometer with Cu Kα as the radiation source (λ = 1.5418 Å, kV = 40, mA = 40) in the 2θ range of 10–90°. The morphology of AuNPs formed in the biopolymer matrix was explored through field emission scanning electron microscopy (FESEM) using a Philips XL30 (Amsterdam, Netherlands) with accelerating voltage 30 kV and 3.0 mm working distance in the secondary electron imaging mode. Furthermore, energy dispersive X-ray spectrometry (EDX) and transmission electron microscopy (TEM, JEOL JEM-1011-instrument) (Tokyo, Japan) were performed. The thermal stability of the samples was tested with the TGA analysis using a Mettler Toledo (Columbus, OH, USA) TGA/SDTA851E instrument by heating the samples at a ramp of 10 °C min^−1^ in the range 50–1000 °C under nitrogen atmosphere. The nitrogen adsorption–desorption isotherms at 77 K were performed using a NOVA 4200e (Quantachrome Instruments, FL, USA). The specific surface area was calculated using the Brunauer–Emmett–Teller (BET) equation.

### 2.3. Catalytic Evaluation of Chitosan-GLA/AuNPs Hybrid Nanocomposite

The catalytic activity of chitosan-GLA/AuNPs nanocomposite was evaluated through the oxidation of benzyl alcohol to its relevant carbonyl compounds, with H_2_O_2_ as an oxidant. The oxidation reaction of alcohol was carried out in a magnetically stirred glass beaker (50 mL). Typically, 1 mmol alcohol substrate, 1.2 mmol H_2_O_2_, and 8.6 mg catalyst were added into 5 mL water. The suspension was then heated up to 80 °C and stirred for 6 h. After completion of the reaction (monitored by TLC), the catalyst was separated from the reaction mixture by centrifuge. The products were analyzed by GC (Perkin Elmer Clarus 500) (Waltham, MA, USA) equipped with a FID detector and identified by comparison with known standards. The recovered catalyst was washed with deionized water and ethanol, dried, and kept in a desiccator. 

## 3. Result and Discussion

### 3.1. FT-IR

**Figure 1:**
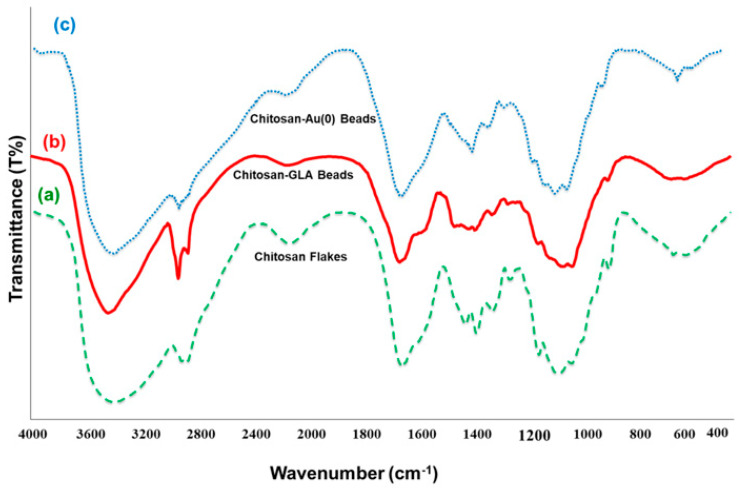
FT-IR of (**a**) chitosan flakes, (**b**) chitosan-GLA beads, and (**c**) chitosan-GLA/AuNPs beads.

The successful cross-linking modification of chitosan and subsequent coordination of Au(III) ions into biopolymer matrix and phytochemical reduction can be partially investigated via FT-IR spectroscopy, as shown in Figure 1. The spectrum of chitosan flakes (Figure 1a) shows a strong, broad vibration at around 3425 cm^−1^ assigned to the stretching vibration of O–H and N–H groups, while the peak at 1650 cm^−1^ is attributed to the N–H bending vibration of primary amine (–NH_2_ in chitosan flakes). After the cross-linking with GLA, this bending band disappeared, due to the participation of –NH_2_ of chitosan in the cross-linking with GLA (Figure 1b). In contrast, the intensity of absorption peaks in the region 2800–3000 cm^−1^, corresponding to the –CH_2_ and –CH_3_ groups of chitosan and GLA, remarkably increased. In addition, a significant new peak in Figure 1b, which appeared at 1662 cm^−1^, is presumably the characteristic band of Schiff base, that is, strong stretching vibration of imine (C=N), confirming the successful cross-linking modification of the chitosan. In addition, the absence of any peak at 1720 cm^−1^ indicated that there was no unreacted pendant aldehyde group and cross-linking was almost completed. 

Due to their excellent chelating capacity, the modified chitosan-GLA beads are considered an attractive adsorbent to coordinate with metal ions. This is mainly because of the large number of hydroxyl functional groups and Schiff-base structure. After adding chitosan-GLA beads to the aqueous solution of HAuCl_4_, the color of the beads gradually changed from orange to dark red as a result of adsorbing Au(III) ions onto the biopolymer matrix. Finally, after mixing these beads with an aqueous solution of *S. argel* leaf extract, the color changed from dark red to black. This is due to the complete reduction of coordinated Au(III) ions to elemental Au and the assembly of AuNPs in the biopolymer matrix. This mechanism was confirmed by the FT-IR and UV–vis spectroscopies analysis. The FT-IR spectrum of chitosan-GLA/AuNPs beads is shown in Figure 1c. The significant difference in FT-IR spectra of the chitosan-GLA and chitosan-GLA/AuNPs beads (curves b and c) was a shift of C=N stretching frequencies from 1662 to 1637 cm^−1^, which is indicative of the back-bonding metal–ligand coordination. The presence of a shoulder at 1550 cm^−1^ is related to the amide II band that may contribute to the amine –NH_2_ group, which was specific for chitosan cross-linking with glutaraldehyde [51,52]. Additionally, the intensity of the O–H stretching band at 3445 cm^−1^ from the hydroxyl groups of chitosan rings decreased when Au loaded to the chitosan chains, suggesting the chelation of Au with both imine and hydroxyl groups of modified chitosan, as shown in Figure 1c. For chitosan-gold beads, the broadening in the region of 2800–3600 cm^−1^ indicates that NH_2_ and OH are involved in the stabilization of AuNPs [36,53]. This result confirms the existence of a chemical bond between the chitosan-GLA matrix and Au nanoparticles.

### 3.2. UV–vis

**Figure 2:**
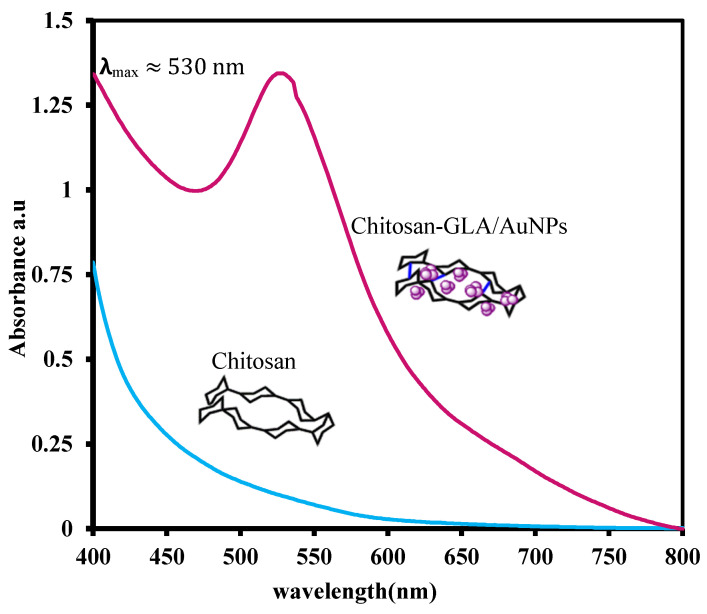
UV–vis absorption spectra of chitosan flakes and chitosan-GLA/AuNPs beads prepared with an aqueous solution of *S. argel* leaf extract.

In addition to the FT-IR evidence, the UV–vis analysis of chitosan flakes and chitosan-GLA/AuNPs beads was helpful to prove the successful assembly of small AuNPs in the polymer matrix. As seen, while the spectrum of chitosan flakes had no absorbance in the region of 400–800 nm, there was a remarkable maximum absorbance (λ_max_) for Au-loaded chitosan at ~530 nm. It has already been shown that the absorbance around ~530 nm is the main characteristic of the gold nanoparticles with core sizes of 2–10 nm in diameter [30,54]. Accordingly, Figure 2 qualitatively indicated the embedment of AuNPs 2–10 nm in the chitosan matrix. It is worth mentioning that a UV–vis analysis of the solution after mixing the chitosan-GLA beads with the aqueous solution of HAuCl4 was performed, and no Au nanoparticle peaks were detected at this stage, confirming that the reduction was caused by the plant extract. 

### 3.3. Elemental Analysis

The elemental analysis of the samples showed that, following the cross-linking modification of chitosan flakes, the N content of samples decreased from 7.80 to 6.95% (Table 1, entries 1 and 2), while an increase in the contents of C and H was observed. This is in good agreement with the attachment of the cross-linking agent (GLA), with several carbon and hydrogen atoms, to the chitosan matrix. In addition, the Au contents of chitosan-GLA/Au(III) and chitosan-GLA/AuNPs beads, estimated by atomic absorption spectroscopy, were 0.1032 and 0.10280 mmol g^−1^, respectively (Table 1, entries 3 and 4). This indicated that ~99% of the coordinated Au(III) ions were phytochemically reduced to elemental gold with *S. argel* leaf extract to form small AuNPs incorporated in the biopolymer matrix.

### 3.4. XRD

**Figure 3:**
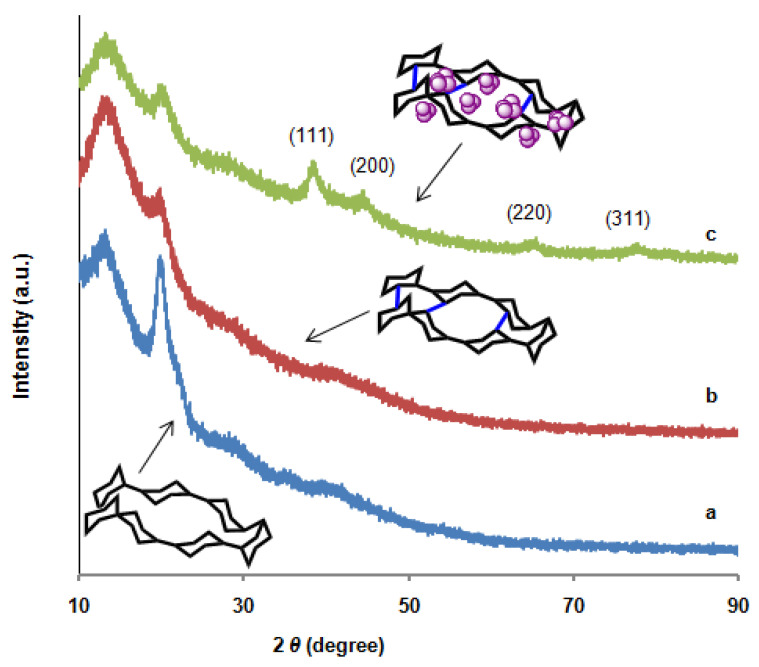
XRD patterns of (**a**) chitosan flakes, (**b**) chitosan-GLA, and (**c**) chitosan-GLA/AuNPs beads.

The growth of AuNPs in the matrix of chitosan-GLA beads under phytochemical reduction of Au(III) ions with *S. argel* leaf extract was also confirmed by XRD analysis. Figure 3a shows the powder XRD patterns for the chitosan flakes and illustrates the presence of two broad peaks observed in the 2*θ* range of 12° to 26° which are assigned to the crystallinity of the original chitosan flakes. As seen in Figure 3b, these peaks are still preserved after the cross-linking process. The characteristic peaks at 38.2°, 44.6°, and 64.6°, attributed to the classical planes of face-centered cubic lattices of AuNPs in chitosan matrix, are also evidenced (Figure 3c), indicating that the embedment of AuNPs in biopolymer networks does not change their crystalline structure. Since the content of AuNPs in the Au–chitosan composite was very low, the diffraction intensities of the gold were weak in the pattern of the composite. According to the full width at half-maximum (FWHM) of the diffraction peak from a crystalline plane (111), the average size of AuNPs in the composite was estimated from the Scherrer equation to be ~8.8 nm. The results were consistent with the electron micrograph observations to be discussed later. Furthermore, the XRD pattern of the samples was used to estimate the degree of crystallinity for each sample using Origin 2020 software. The results of crystallinity are recorded in Table 2, which shows that chitosan-GLA/AuNPs beads have the highest degree of crystallinity of 64.21%.

### 3.5. FESEM

**Figure 4:**
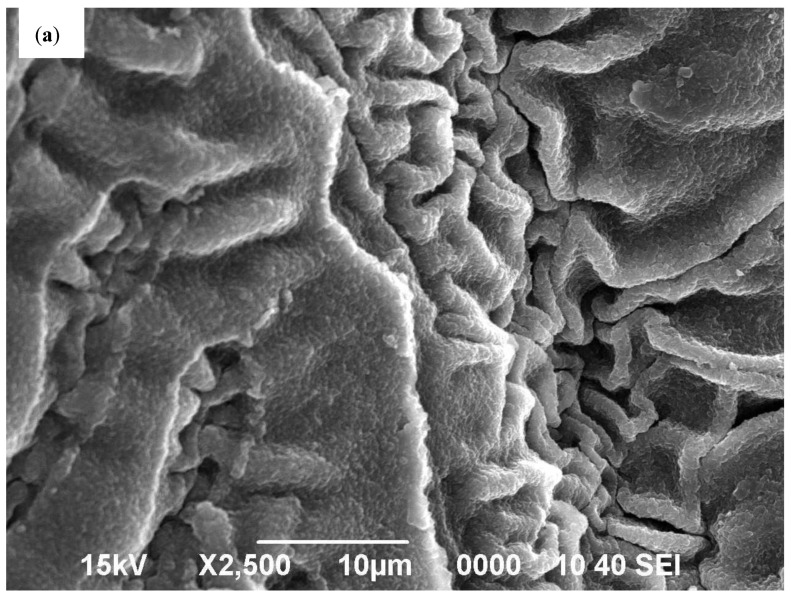

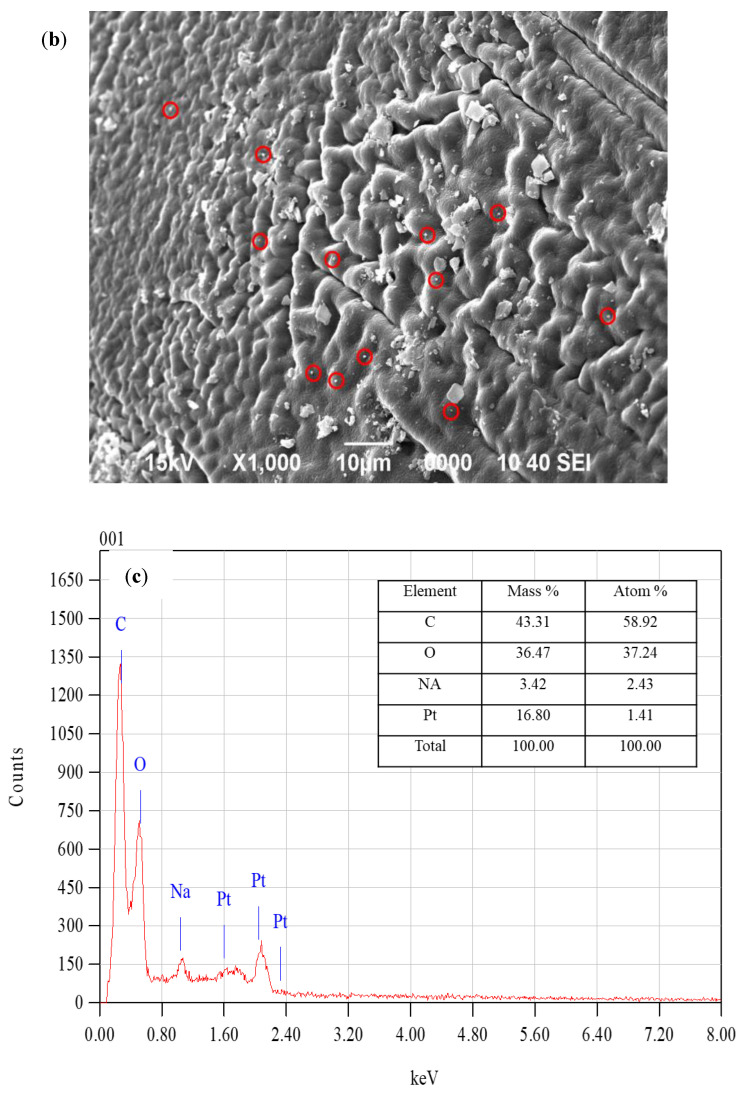

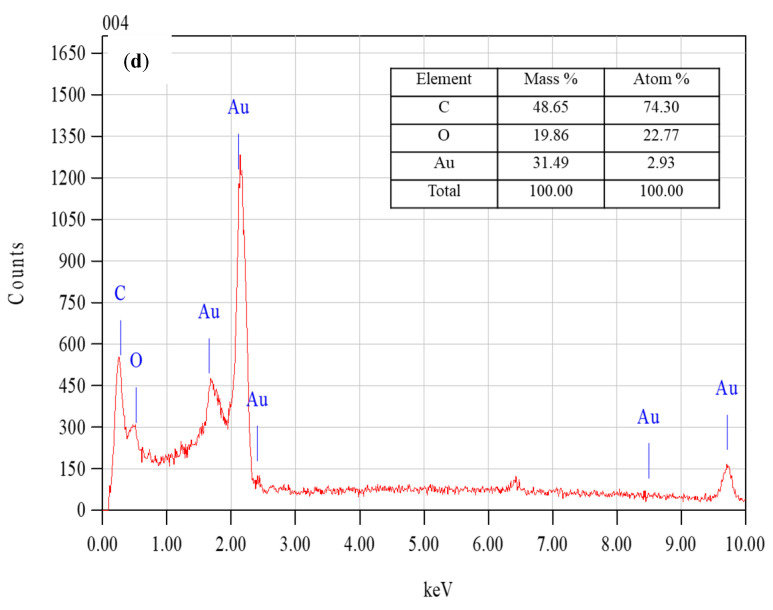
FESEM images (**a**,**b**) and EDS (**c**,**d**) of chitosan-GLA and chitosan-GLA/AuNPs beads, respectively.

Figure 4 shows the FESEM images of the chitosan-GLA (a) and chitosan-GLA/AuNPs (b). As seen, some spherical Au particles were detected on the surface of the chitosan (surrounded by red circles in Figure 4b). However, most of the AuNPs are mostly encapsulated within the chitosan polymeric matrix, due to the cross-linking modification process. Figure 4c,d shows the EDS analysis, which confirms that chitosan-GLA beads contain no Au atom, while the chitosan-GLA/AuNP nanocomposite contains about 31.49 wt.% Au (2.93 atom%), 48.65 wt.% carbon (74.30 atom%), and 19.86 wt.% oxygen (22.77 atom%), as shown in the insert of Figure 4c,d. Since no other capping or reducing agents were used during synthesis, these results thus clearly demonstrated that *S. argel* leaf extract acted as a reducing agent. On the other hand, the chitosan-GLA beads acted as a sorbent to coordinate with Au(III) ions and a dispersant to trap the initially formed gold seeds and prevent them from further growth and aggregation in the synthesis process of AuNPs. The appearance of some Pt traces in the chitosan-GLA beads analysis (Figure 4c) is related to the platinum grid used as a sample holder.

### 3.6. TGA

**Figure 5:**
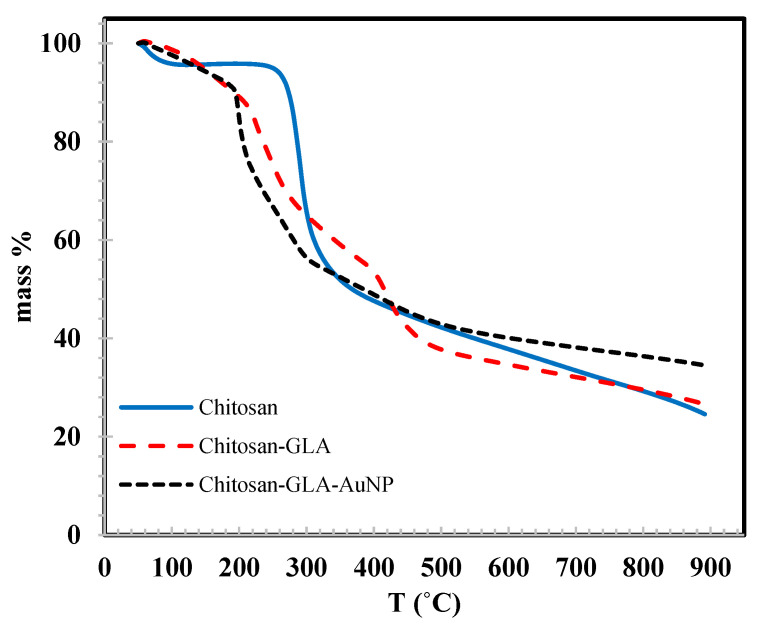
TGA thermograms of chitosan, chitosan-GLA, and chitosan-GLA/AuNPs composites.

The thermal stability of the prepared nanocomposites was explored by the thermogravimetric analysis (TGA) of chitosan, chitosan-GLA, and chitosan-GLA/AuNPs. Figure 5 shows the three stages of mass loss for chitosan. The first loss is attributed to the loosely bonded moisture (~5%) in the range 50–100 °C. The chitosan mass was then stable until the beginning of the second stage at 240 °C. The second weight loss (~41%) occurred in the range of 240–330 °C, due to the decomposition of polymer chains. The third stage is slower than the second and is extended in the temperature range 330–900 °C, with a loss of ~27% related to the continuous decomposition of the polymer chain. The total chitosan loss for the entire temperature range was 73%. The chitosan-GLA/AuNPs and the chitosan-GLA follow the same profile for the first stage in the temperature range 50–195 °C, with a mass loss of ~10%, which may be related to the loss of moisture attached to the surface. After 195 °C, the mass loss of chitosan-GLA was slower than that of chitosan-GLA/AuNPs until 430 °C, with a total loss of 54% for the three samples. The loss in this stage could be attributed to the decomposition of chitosan and GLA chains in addition to *S. argel* leaf extract coated on AuNPs for chitosan-GLA/AuNP. The third stage is in the range 430–900 °C, with mass losses of 12%, 19%, and 19% for chitosan-GLA/Au, chitosan-GLA, and chitosan-GLA, respectively, which may be related to the decomposition of chitosan, GLA, and *S. argel* leaf extract. The total mass loss of chitosan-GLA/Au (65%) was lower than that of chitosan and chitosan-GLA (73%), due to the strengthening effect of the embedded AuNPs, which is believed to play a key role in composite stability. It is assumed that AuNPs with higher surface energy simply combine with the oxygen or water in the air and increase the possibility for interfacial hydrogen-bonding formation. In consequence, this could reduce the mobility of chitosan chains in the composite. The chain mobility reduction could in turn suppress the chain transfer reaction and slow the degradation process of the biopolymer, and so its decomposition could take place at higher temperatures. These results demonstrated that the thermal stability of chitosan remarkably improved when modified with GLA and doped with AuNPs. 

### 3.7. TEM

**Figure 6:**
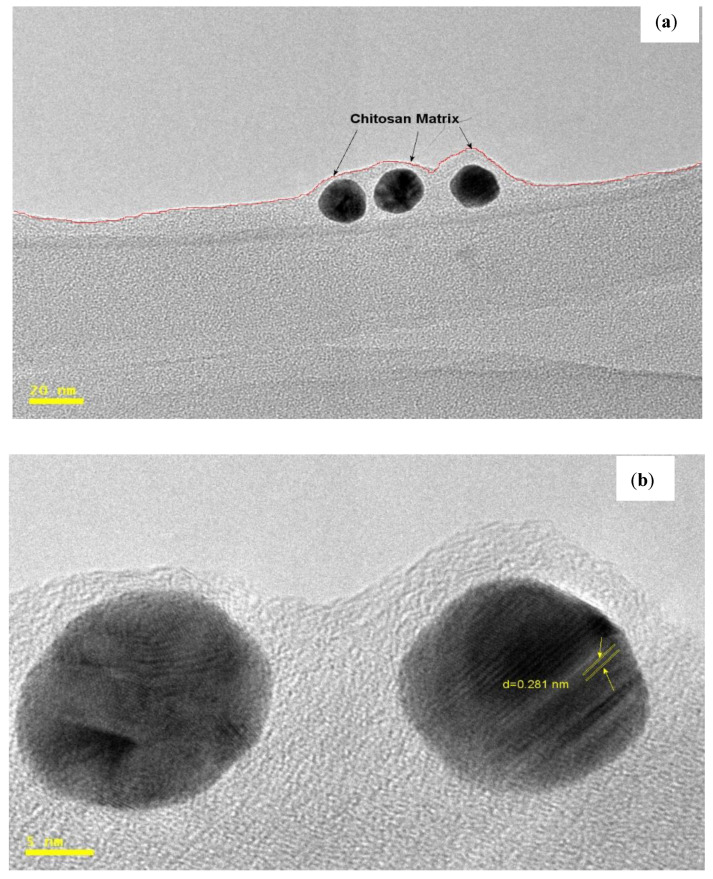

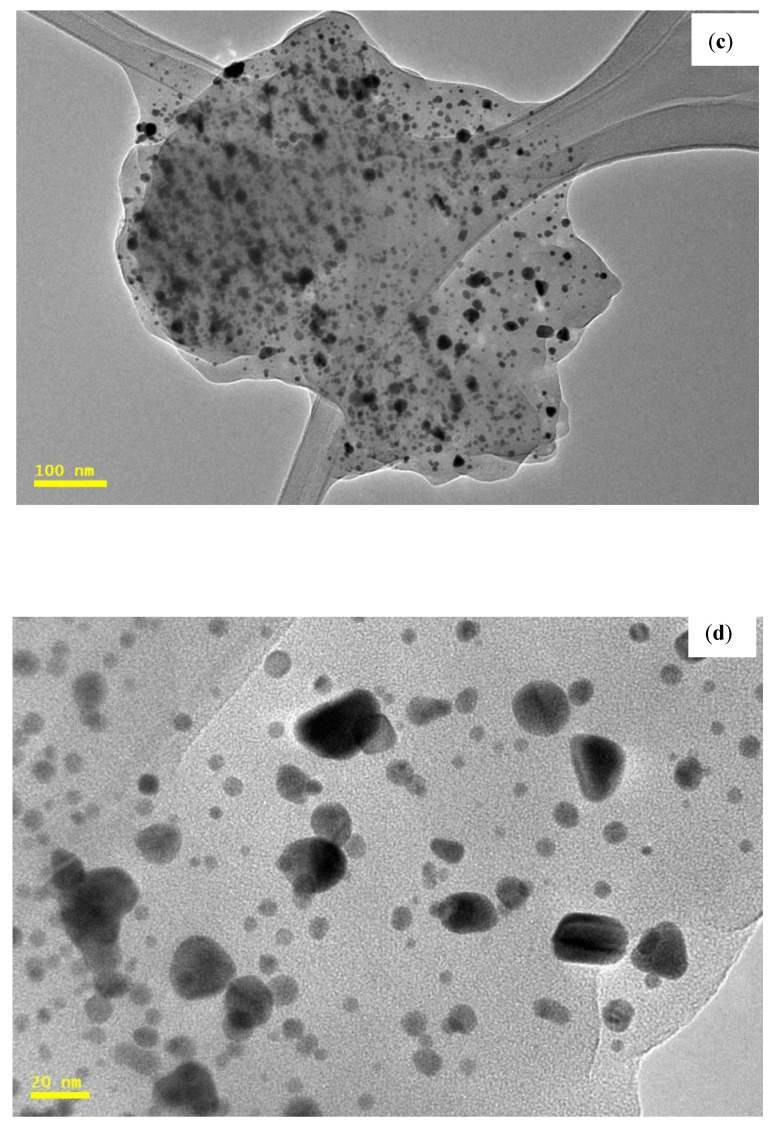

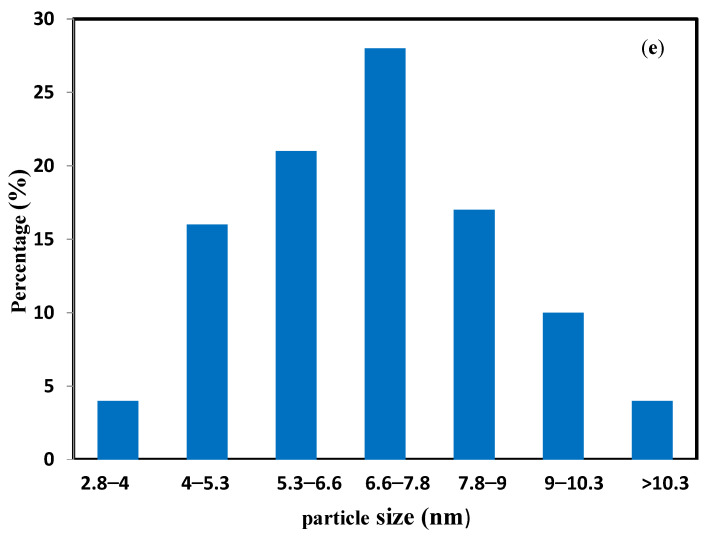
HRTEM images of chitosan-GLA/AuNPs nanocomposite (**a**); the lattice fringes in the (1 1 1) plane of the particles and measured lattice *d*-spacing (**b**); TEM image of AuNPs prepared with *S. argel* leaf extract without using cross-linked chitosan (**c**); TEM image of AuNPs prepared with *S. argel* leaf extract using cross-linked chitosan (**d**); and the particle size distributions of AuNPs within the chitosan-GLA/AuNPs nanocomposite (**e**).

The transmission electron microscopy imaging provided further insight into the morphology and mean size of the AuNPs loaded in the chitosan matrix. A representative TEM image recorded from chitosan-GLA/AuNPs beads synthesized using *S. argel* leaf extract is shown in Figure 6a. As seen in the image, the spherical AuNPs with diameters no more than 10 nm are well separated and reasonably dispersed. Figure 6b shows single-crystal AuNPs with a lattice *d*-spacing of 2.36°A in the (1 1 1) plane, which is related to face-centered cubic (f c c) structure. Additionally, to understand the role of chitosan in the phytochemical-assisted formation of AuNPs, in a control experiment the possibility of the formation of AuNPs using *S. argel* leaf extract without cross-linked chitosan was tested. The TEM image of the product (Figure 6c) showed that there was a wide range of sizes and different morphologies and shapes of the AuNPs (spherical, triangle, cubic, and oval), and various sizes were observed. This revealed that, even though the *S. argel* leaf extract is capable of reducing the Au(III) ions to elemental Au, using cross-linked chitosan is necessary to have better control over the shape and size. Huang et al. (2007) prepared triangular and spherical gold nanoparticles by reacting *Cinnamomum camphora* leaf extract with aqueous gold precursors. They attributed the controlled shape of the gold nanoparticles to the protective and reductive biomolecules used [55]. Dwivedi et al. (2010) used *Chenopodium album* leaf extract for the green synthesis of gold nanoparticles and reported 10–30 nm spherical nanoparticles [43]. Sugunan et al. (2005) prepared spherical gold nanoparticles with an average size of 20 nm supported on chitosan [56]. This controlling effect of chitosan on morphology and size of the AuNPs is due to the lowering of the growth rate of initially formed small spherical Au seeds, which are trapped in a polymeric matrix (Figure 6a), show no tendency to aggregate, and are stable for one year. The image in Figure 6d was used in Figure 6e about the particle size distribution histogram. Accordingly, the average particle size of AuNPs was found to be around 6.0 ± 3 nm, which is consistent with the results obtained by XRD (8.8 nm).

Altogether, the aforementioned spectroscopic and microscopic observations confirmed the phyto-inspired assembly of Au atoms in the matrix of cross-linked chitosan with the aid of *S. argel* leaf extract, resulting in the feasible formation of highly stable spherical AuNPs smaller than 10 nm. All processes occurred at room temperature and were completed in a few minutes through a green and cost-effective route without using any organic solvent or chemical toxic reducing/stabilizing agents. 

### 3.8. Specific Surface Area (BET)

BET surface area for the chitosan at different stages of modification was measured and recorded in Table 2. The BET surface area of the chitosan-GLA/AuNPs beads is 4.90 m^2^g^−1^, which is better than many other modified chitosans reported in the literature [57]. This relatively high specific surface area for the chitosan-GLA/AuNPs beads is useful for the catalytic oxidation activity of this catalyst. It is apparent in Table 2 that the sample with the highest surface area resulted in the highest oxidation activity. The BET results strongly support the fact that the chitosan-GLA/AuNPs have a porous structure, as indicated by SEM [57]. 

### 3.9. Catalytic Activity Studies

The catalytic activity of the AuNPs loaded in the chitosan matrix was quantitatively determined for the oxidation of benzyl alcohol (BnOH) by H_2_O_2_ to its relevant carbonyl compounds. This process is a very important intermediate in the production of various biologically and industrially important substances. It was observed that in the presence of a specific amount of chitosan-GLA/AuNPs beads, with Au content of 0.102 mmol, stirring of the reaction medium containing an aqueous solution of BnOH and H_2_O_2_ led to the desired benzaldehyde compounds as final products. For instance, it was found that a combination of 1 mmol of BnOH, 1.25 mmol of H_2_O_2_, and 8.6 mg of chitosan-GLA/AuNPs beads and stirring the mixture for 6 h at 80 °C could result in the remarkable conversion (97%) and excellent selectivity (99%) in the formation of benzaldehyde over the other possible products. 

It is worth mentioning that, while in the positive control experiment, in the absence of a catalyst, only 10% conversion of BnOH was observed (Table 2, entry 2), in the negative control experiment with chitosan-GLA/AuNPs catalyst, in the absence of H_2_O_2_, the solution remained unchanged. Meanwhile, using *S. argel* leaf extract, chitosan flakes, chitosan-GLA beads, and chitosan-GLA/Au^3+^ beads as catalysts showed very low to moderate conversions (Table 2, entries 3–6). As shown, none of these added compounds could efficiently catalyze the oxidation reaction. The moderate conversion observed using chitosan-GLA/Au^3+^ beads was due to Au^3+^-promoted oxidation of BnOH. Altogether, these simple control experiments confirmed the efficient catalytic role of chitosan-GLA/AuNPs composite in BnOH oxidation with excellent conversion. In addition, the Au-chitosan hybrid nanocatalyst was found to be reusable at least for four runs (Table 2, entries 7–9), and during the reuse experiments, no deformation, agglomeration, or Au leaching was observed. 

## 4. Conclusions

In summary, a novel, green, and cost-effective combination of cross-linked chitosan and biological leaf extract of a plant (*S. argel*) has been utilized towards the assembly of stable AuNPs in the matrix of a biopolymer. The cross-linking of chitosan with glutaraldehyde provided a thermally stable framework for loading of Au(III) ions and their reduction to elemental Au by *S. argel* leaf extract, instead of any other toxic chemical reducing agent. The processes in this study provided a method for the preparation of well-controlled size and morphology of gold nanoparticles and may find significant industrial importance in solid-supported metal nanoparticles preparation. The Au particles were spherical and monodispersed (<10 nm). In addition, the catalytic performance of the obtained Au-chitosan hybrid nanocomposite was evaluated. The resultant nanocomposite showed remarkable performance of catalytic activity towards the oxidation reaction of benzyl alcohol to its relevant carbonyl compounds with excellent conversion and selectivity, using hydrogen peroxide as an oxidant. Moreover, the recyclability features of this nanocomposite make it a good candidate to be considered as a green catalyst in bioorganic synthetic routes to drugs and pro-drugs.

## Figures and Tables

**Table 1:** Elemental analysis of chitosan flakes and modified chitosan with GLA, Au^3+^, and AuNPs.

Entry	Samples		Elements			
		C%	%H	%N	Au	
					% atom	mmol g^−1^
1	Chitosan	40.91	7.54	7.80	-	-
2	Chitosan-GLA	43.58	7.81	6.95	-	-
3	Chitosan-GLA/Au^3+^	51.59	8.81	5.52	2.033	0.103
4	Chitosan-GLA/AuNPs	51.63	8.83	5.50	2.024	0.102

**Table 2:** BET specific surface area, degree of crystallinity, and the catalytic oxidation of benzyl alcohol with H_2_O_2_ in the presence of various compounds.

Entry	Catalyst	BET (m^2^g^−1^)	Crystallinity (%)	Conversion % ^a^
1	chitosan-GLA/AuNPs beads	4.90	64.21	97.5
2	No catalyst	-	-	10.0
3	*S. argel* leaf extract	-	-	12.8
4	chitosan flakes	3.25	62.03	4.3
5	chitosan-GLA beads	4.73	63.18	14.9
6	chitosan-GLA/Au^3+^ beads	-	-	42.3
7	chitosan-GLA/AuNPs beads			95.1 ^b^
8	chitosan-GLA/AuNPs beads			91.3 ^c^
9	chitosan-GLA/AuNPs beads			70.0 ^d^

^a^ Determined by GC. ^b–d^ 2nd, 3rd, and 4th run, respectively.

## Data Availability

The data presented in this study are available on request from the corresponding author.
